# Supplementary material for: Legacies of domestication, Neolithic diffusion and trade between Indian subcontinent and Island Southeast Asia shape maternal genetic diversity of Andaman cattle
Source: PLoS One. 2022 Dec 9;17(12):e0278681. doi: 10.1371/journal.pone.0278681 (PMC9733863; doi:10.1371/journal.pone.0278681)
Supplement: S1 Table — (DOCX) [file pone.0278681.s001.docx]

**S1 Table. Details of sampling location of Andaman cattle (ANC)**

| **S. No.** | **Name of the Village** | **Latitude** | **Longitude** | **Samples (n)** |
| --- | --- | --- | --- | --- |
| **North and Middle Andaman (n=80)** | | | |  |
| 1 | Shyam Nagar | 13.3940327°N | 92.9329579°E | 3 |
| 2 | Madhupur | 13.2634408°N | 92.9655987°E | 3 |
| 3 | Durgapur | 13.2709482°N | 93.031836°E | 3 |
| 4 | Shibpur | 13.2429957°N | 93.0292434°E | 3 |
| 5 | Khudirampur | 13.2353575°N | 92.9658488°E | 3 |
| 6 | Nischintapur | 13.2126924°N | 92.87134°E | 3 |
| 7 | Kishorinagar | 13.1619115°N | 92.8785916°E | 3 |
| 8 | Nabagram | 13.1622309°n | 92.9413319°E | 3 |
| 9 | Kalighat | 13.1173453°N | 92.9607206°E | 3 |
| 10 | Subashgram | 13.2470781°N | 92.9713627°E | 3 |
| 11 | Swarajgram RV | 13.3445257°N | 92.9366375°E | 3 |
| 12 | Laxmipur | 13.2941301°N | 92.9560698°E | 3 |
| 13 | Sitanagar | 13.2311479°N | 92.9453471°E | 3 |
| 14 | Webi | 12.8377486°N | 92.8879944°E | 3 |
| 15 | Lucknow | 12.8528653°N | 92.8760396°E | 3 |
| 16 | Pahalgaon RV | 12.8363417°N | 92.8672744°E | 3 |
| 17 | Devpur | 12.851413°N | 92.8778424°E | 3 |
| 18 | Betapur | 12.717003°N | 92.8967461°E | 3 |
| 19 | Billiground | 12.6660062°N | 92.8861427°E | 3 |
| 20 | Kaushalyanagar RV | 12.539224°N | 92.8253046°E | 3 |
| 21 | Tugapur | 12.8382963°N | 92.8508022°E | 2 |
| 22 | Govindapur | 12.7169142°N | 92.8868784°E | 2 |
| 23 | Parnasala | 12.5173105°N | 92.9109817°E | 2 |
| 24 | Bakultala | 12.5039982°N | 92.8624749°E | 2 |
| 25 | Nimbutala RV | 12.6830231°N | 92.8999739°E | 2 |
| 26 | Desharatpur RV | 12.5070085°N | 92.9253619°E | 2 |
| 27 | Long Island | 12.3969966°N | 92.9392333°E | 2 |
| 28 | Kadamtala | 12.3460107°N | 92.7760395°E | 2 |
| 29 | Nilambur | 12.1755603°N | 92.7874718°E | 2 |
| 30 | mayabunder | 12.9154241°N | 92.9023218°E | 2 |
| **South Andaman (n=50)** | | | |  |
| 1 | Ferrargunj | 11.7196877°N | 92.6560736°E | 3 |
| 2 | Burmanallah | 11.5662657°N | 92.7335091°E | 3 |
| 3 | Wandoor | 11.5977535°N | 92.6200246°E | 3 |
| 4 | Manjery | 11.5429345°N | 92.6531876°E | 3 |
| 5 | Guptapara | 11.5645565°N | 92.6586968°E | 3 |
| 6 | Wimberlygunj | 11.7372555°N | 92.7070383°E | 3 |
| 7 | Tushnabad | 11.6759344°N | 92.6445606°E | 3 |
| 8 | Colinpur RV | 11.6929167°N | 92.6207657°E | 3 |
| 9 | Chouldari | 11.6590383°N | 92.667286°E | 3 |
| 10 | Manpur RV | 11.6774373°N | 92.6535479°E | 3 |
| 11 | Ograbraij RV | 11.6698675°N | 92.6665149°E | 2 |
| 12 | Bambooflat | 11.702946°N | 92.7140279°E | 2 |
| 13 | Stweartgunj RV | 11.7246541°N | 92.7071558°E | 2 |
| 14 | Manglutan | 11.5826195°N | 92.6603712°E | 2 |
| 15 | Brichgunj | 11.6169487°N | 92.7466193°E | 2 |
| 16 | Brookshabad | 11.6378244°N | 92.7392592°E | 2 |
| 17 | Calicut | 11.5987679°N | 92.714457°E | 2 |
| 18 | Chidiyatapu | 11.5141903°N | 92.7002944°E | 2 |
| 19 | Rangachang | 11.5833359°N | 92.7346006°E | 2 |
| 20 | Sippighat | 11.6181493°N | 92.7124335°E | 2 |
| **Nicobar (n=20)** | | | |  |
| 1 | Big Lapathy | 9.2303499°N | 92.7894953°E | 3 |
| 2 | Kinyuka | 9.2078549°N | 92.8147275°E | 3 |
| 3 | Kinmai | 9.2129308°N | 92.7787135°E | 3 |
| 4 | Tamaloo | 9.1905646°N | 92.8148175°E | 3 |
| 5 | Malacca | 9.1776242°N | 92.8166597°E | 2 |
| 6 | Perka | 9.1776242°N | 92.8166597°E | 2 |
| 7 | Sawai | 9.2190516°N | 92.7176187°E | 2 |
| 8 | Kakana | 9.1598133°N | 92.8002774°E | 2 |
