# Supplementary material for: Legacies of domestication, Neolithic diffusion and trade between Indian subcontinent and Island Southeast Asia shape maternal genetic diversity of Andaman cattle
Source: PLoS One. 2022 Dec 9;17(12):e0278681. doi: 10.1371/journal.pone.0278681 (PMC9733863; doi:10.1371/journal.pone.0278681)
Supplement: S2 Table — (DOCX) [file pone.0278681.s002.docx]

**S2 Table. GenBank Accession numbers used in the study**

| **Country** | **Accession No.** | **Haplogroups** |
| --- | --- | --- |
| Vietnam | JX040452-JX040457, JX040458-JX040469 | I1=16  I2=2 |
| Thailand | HM173342-HM173351, MH746114-MH746120 | I1=6  I2=11 |
| Philippines | MH638815, MH638817, MH638820-MH638821, MH638823, MH638827-MH638828, MH638832,  MH638835-MH638836, MH638838-MH638839,  MH638842, MH638849, MH638857-MH638858,  MH638862-MH638864, MH638866-MH638867,  MH638869-MH638870, MH638872-MH638878,  MH638880-MH638885, MH638889-MH638890,  MH638893-MH638894, MH638899, MH638901,  MH638903-MH638904, MH638908-MH638909,  MH638911-MH638912, AB079325-AB079326 | I1=45  I2=5 |
| Pakistan | JF825055-JF825058, JF501217 | I2=5 |
| Nepal | AB065119-AB065131, AB570166-AB570175  AB085918-AB085921 | I1=3  I2=9  T=15 |
| Myanmar | LC377275-LC377280, LC377282-LC377301 | I1=17  I2=9 |
| Bhutan | AB570113- AB570165, AB268559-AB268584 | I1=36  I2=30  T=13 |
| Bangladesh | KY682307-KY682323 | I1=14  I2=3 |
| Central India | KP223261- KP223272, GQ890024, GQ890046-GQ890047, GQ890096-GQ890112 | I1=17  I2=12  T=3 |
| Eastern India | KP223257-KP223260, KP223273-KP223282,  KU682457-KU682490, JN417003 | I1=17  I2=26  T=6 |
| Southern India | GQ890017, GQ890019, GQ890020-GQ890023,  GQ890025-GQ890030, GQ890054, GQ890057,  GQ890074-GQ890081, GQ890093-GQ890095, GQ890113-GQ890114, GQ890115, GQ890117,  GQ890118-GQ890119, GQ890144-GQ890149  AY378133-AY378134, AY378135-AY378136,  AF336738-AF336742 | I1=23  I2=18  T=5 |
| Western India | GQ890016, GQ890018, GQ890031-GQ890045, GQ890048-GQ890053, GQ890055-GQ890056, GQ890058-GQ890073, GQ890082-GQ890092,  GQ890116, GQ890120-GQ890143, HQ234718-HQ234743, U51806-U51815, AB085922, AB085923, JF825058, AY378137 | I1=64  I2=43  T=9 |
