# Supplementary material for: Legacies of domestication, Neolithic diffusion and trade between Indian subcontinent and Island Southeast Asia shape maternal genetic diversity of Andaman cattle
Source: PLoS One. 2022 Dec 9;17(12):e0278681. doi: 10.1371/journal.pone.0278681 (PMC9733863; doi:10.1371/journal.pone.0278681)
Supplement: S3 Table — (DOCX) [file pone.0278681.s003.docx]

**S3 Table**. **Haplogroup assignment of Andaman cattle haplotypes**

| **Haplogroups** | **Andaman cattle haplotypes** |
| --- | --- |
| I1 haplogroup | ANCHT2, ANCHT3, ANCHT5, ANCHT8, ANCHT10, ANCHT13, ANCHT14, ANCHT17, ANCHT20, ANCHT22, ANCHT23, ANCHT24, ANCHT25, ANCHT26, ANCHT27, ANCHT28, ANCHT29, ANCHT30, ANCHT31, ANCHT32, ANCHT33, ANCHT34, ANCHT35, ANCHT36, ANCHT37, ANCHT38, ANCHT40, ANCHT41, ANCHT42, ANCHT43, ANCHT45, ANCHT46, ANCHT47, ANCHT48, ANCHT49, ANCHT51, ANCHT52, ANCHT53, ANCHT56, ANCHT57, ANCHT58, ANCHT59, ANCHT61, ANCHT63, ANCHT64, ANCHT65, ANCHT66, ANCHT68, ANCHT69, ANCHT70, ANCHT71, ANCHT72, ANCHT73, ANCHT75, ANCHT76, ANCHT79, ANCHT80, ANCHT81 |
| I2 haplogroup | ANCHT1, ANCHT4, ANCHT6, ANCHT7, ANCHT11, ANCHT12, ANCHT16, ANCHT18, ANCHT19, ANCHT21, ANCHT39, ANCHT44, ANCHT50, ANCHT54, ANCHT55, ANCHT60, ANCHT67, ANCHT74, ANCHT77, ANCHT78 |
| T/Q haplogroup | ANCHT9, ANCHT15 and ANCHT62 |
